# Optogenetic approach for targeted activation of global calcium transients in differentiated C2C12 myotubes

**DOI:** 10.1038/s41598-017-11551-z

**Published:** 2017-09-11

**Authors:** Stéphane Sebille, Oualid Ayad, Charles-Albert Chapotte-Baldacci, Christian Cognard, Patrick Bois, Aurélien Chatelier

**Affiliations:** Laboratoire de Signalisation et Transports Ioniques Membranaires, Université de Poitiers, CNRS, 86073, Poitiers, CEDEX 9 France

## Abstract

Excitation-contraction coupling in muscle cells is initiated by a restricted membrane depolarization delimited within the neuromuscular junction. This targeted depolarization triggers an action potential that propagates and induces a global cellular calcium response and a consequent contraction. To date, numerous studies have investigated this excitation-calcium response coupling by using different techniques to depolarize muscle cells. However, none of these techniques mimic the temporal and spatial resolution of membrane depolarization observed in the neuromuscular junction. By using optogenetics in C2C12 muscle cells, we developed a technique to study the calcium response following membrane depolarization induced by photostimulations of membrane surface similar or narrower than the neuromuscular junction area. These stimulations coupled to confocal calcium imaging generate a global cellular calcium response that is the consequence of a membrane depolarization propagation. In this context, this technique provides an interesting, contactless and relatively easy way of investigation of calcium increase/release as well as calcium decrease/re-uptake triggered by a propagated membrane depolarization.

## Introduction

Motor neuron inputs trigger electrical activity of muscle fibers through the neuromuscular junction^1–3^. Locally, activation of postsynaptic nicotinic receptors induces membrane depolarization and the subsequent action potential propagation to trigger intracellular calcium increase and cell contraction^4, 5^. As a consequence, such a system ensures the fast membrane depolarization and associated rise in calcium in the entire cell in response to the stimulation of a restricted membrane area delimited by the neuromuscular synapse^6^.

Numerous studies have investigated calcium homeostasis in developing skeletal muscle cells^7–9^ and, depending on their maturation state, the excitation/calcium release coupling displays some differences. In non-mature cells, myoplasmic calcium increases income from both extracellular calcium entries through voltage-gated calcium channels and from sarcoplasmic reticulum releases^10^ whereas in mature myotubes, this mainly occurs through calcium release from sarcoplasmic reticulum^11^. Given the essential role of calcium in muscle function, numerous pathologies are related to dysregulations of calcium homeostasis such as Duchenne muscular dystrophy^11, 12^. In this context, excitation-calcium release coupling in muscle cell is widely studied using different techniques to induce cell depolarization with associated intracellular calcium variation.

To date, several methods are commonly used to investigate excitation/calcium release coupling *in vitro*: patch-clamp technique, stimulation by means of an electric field using external electrodes^13–15^, superfusion of a depolarizing high-concentrated potassium solution^11, 12, 16^ and focal pressure application (puff) of acetycholine through a glass capillarie^17–19^. However, most of these techniques display some weaknesses in their spatial and temporal resolution of cell depolarization properties. The common observed drawback is a weak spatial resolution since the techniques cited above necessarily stimulate a large area containing numerous cells or a large part of the cell. This does not allow the specific excitation of one cell or of a small-restricted area within this cell as observed in the neuromuscular junction. Besides, stimulation of muscle cells through electrodes depends on non-uniform electrical field spaces and results in the production of toxic gases and alterations of pH limiting long lasting depolarizations^20^. In addition, stimulation with a high extracellular potassium solution depends on the superfusion kinetics (and spatial diffusion), which is very difficult to control and provides longer depolarization as compared to action potentials generated through the activation of a motor end-plate. This low temporal and spatial resolutions do not allow studying precisely both intracellular calcium rise and decrease initiated by membrane depolarization. Therefore, these techniques are limited for the study of fast transient cellular/subcellular calcium concentration variations in response to action potentials as they fail to mimic localized APs generation at the post-synaptic membrane.

Since it has been first described and developed in neural cells^21^, optogenetics has spurred immense research activity. This emerging technology has allowed huge improvement in the understanding of neural circuitry and brain function by the optical interrogation with high specificity and high spatiotemporal resolution of the electrical activity of targeted neurons. Whereas optogenetics tools appear to be now appropriately mastered, they are still very poorly used in muscular research. Thus, it seems of particular interest to apply these new tools and methods in combination with electrophysiology, biochemistry and molecular biology approaches, to assess physiology and physiopathology of striated muscles. It has recently been demonstrated that muscular contraction was optically controlled in a synchronous manner using a given train of light pulses when a myotube was generated from C2C12 clonal myoblasts, which were genetically engineered to express channelrhodopsin-2 (ChR2)^22^. These contractions were the consequences of light-evoked membrane depolarization. Moreover, another study used optogenetic stimulation to induce contraction of intact skeletal muscle of transgenic mice^23^. However, the relationship between optical stimulation and calcium homeostasis in these cells has never been investigated yet.

In this work, using highly localized short spots of light in ChR2 expressing C2C12 myotubes, the kinetics properties of intracellular calcium increases were investigated. This enabled us to analyze calcium variations in response to membrane depolarization with fine temporal and spatial resolution. This contactless manner to activate skeletal muscle cells was found to be reliable in space and time and provides a powerful tool to model “motor neuron inputs”- like activation.

## Results

### Expression of ChR2-GFP in C2C12 myotubes

**Figure 1:**
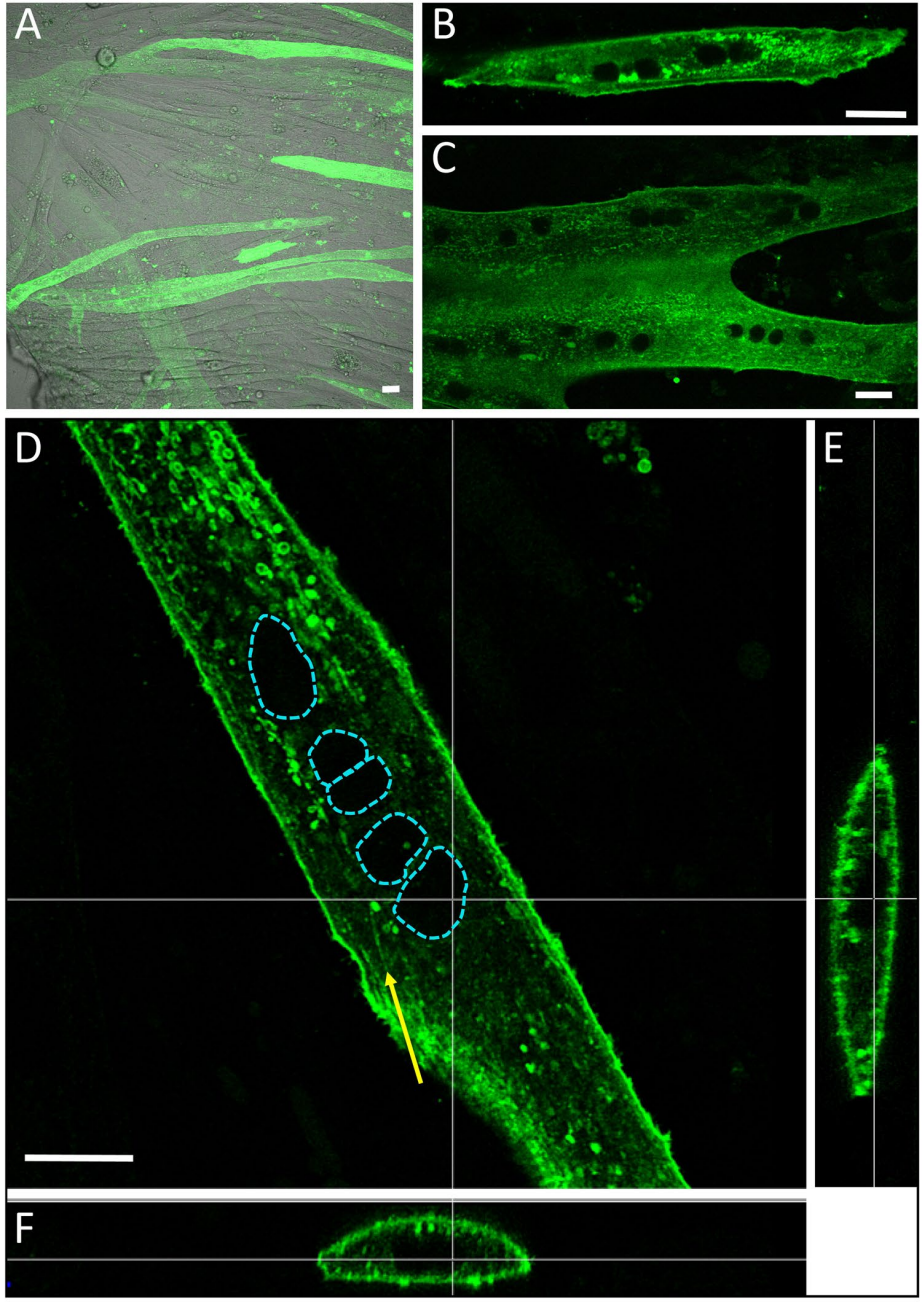
(**A**) Confocal imaging of ChR2-GFP expressing myotubes at D7: ChR2-GFP positive myotubes display a bright green fluorescence. (**B**) A ChR2-GFP expressing myotube at D4. (**C**) A ChR2-GFP expressing myotube at D12, (**D**,**E**,**F**) 3D expression pattern of ChR2 in a myotube at D10. Yellow arrows: putative T-Tubule formation structures. The dashed blue lines mark out nuclear areas. Bars: 15 µm.

ChR2-GFP protein was expressed in C2C12 cells using cellular transfection of pAAV-CAG-ChR2-GFP. The differentiation process was initiated twenty-four hours after transfection and multinuclear myotubes were obtained after 3 to 4 days of fusion. Efficient expression of ChR2 was checked by GFP fluorescence (Fig. 1). In our conditions, ChR2-GFP positive myotubes were easily discriminated from the non-fluorescent ones and represent around 15 to 25% of total multinucleated myotubes at D7 (Day 7, Fig. 1A). ChR2-GFP expressing myotubes displayed a bright fluorescence from D4 (Fig. 1B) to D12 (Fig. 1C). Z-slices confocal imaging allowed us to explore the 3D expression pattern of ChR2 in C2C12 myotubes. Despite the presence of intracellular green fluorescent spots distinct from mitochondria (see Fig. S1), Channel-Rhodopsin-2 is largely expressed within the sarcolemma (Fig. 1D–F) and possibly in tubules in formation at this stage of differentiation (Fig. 1D: yellow arrow).

### Functional expression of ChR2 in C2C12 myotubes

**Figure 2:**
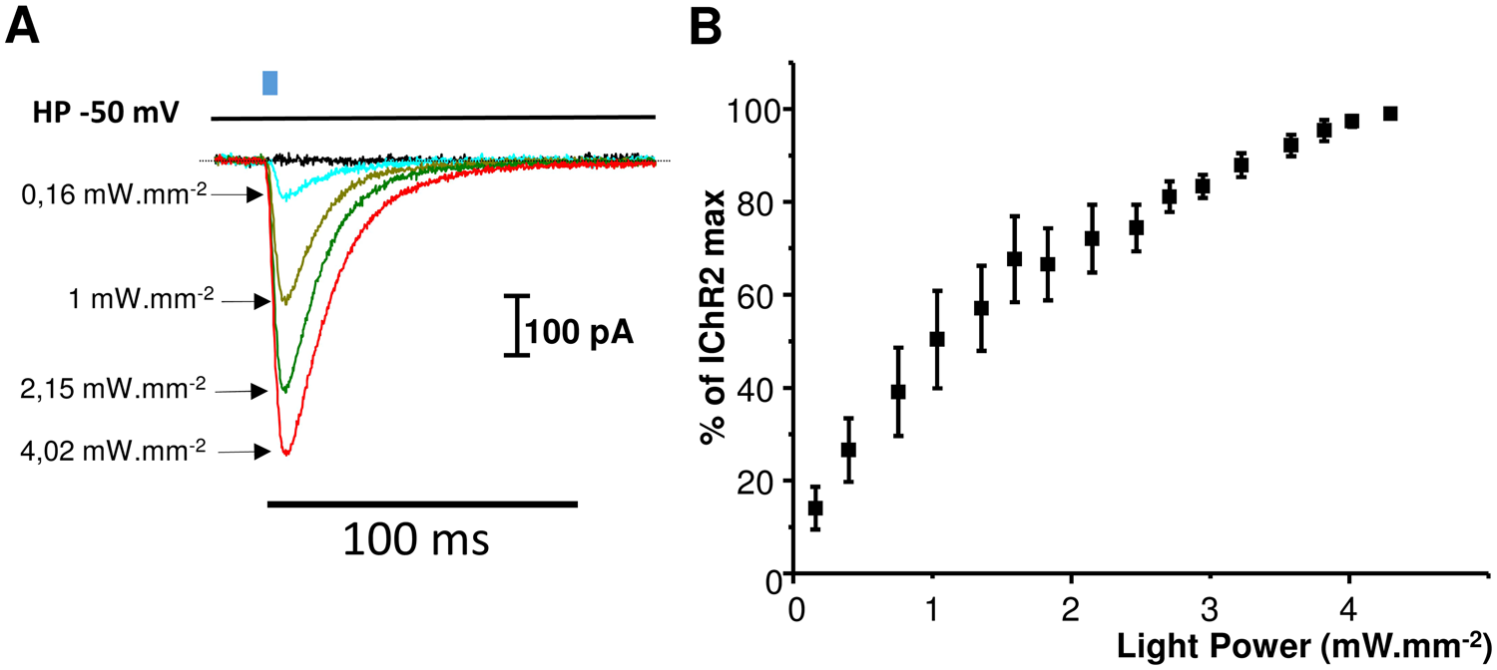
Functional expression of ChR2 in C2C12 myotubes investigated by patch-clamp. (**A**) Inward ChR2 current recorded at a holding potential of −50 mV during a light pulse of 4 ms at various light intensities. Light pulse is schematized as a blue rectangle. (**B**) ChR2 current recorded as described in A represented as percentage of maximum ChR2 current recorded at 4.3 mW.mm^−2^. Data are expressed as mean ± SEM (n = 6).

In our experiments, C2C12 myotubes displayed a resting membrane potential of −50.5 ± 0.8 mV (n = 6). The functionality of ChR2 in C2C12 myotubes was assessed to evaluate its ability to be activated by 4 ms light pulses. Shedding 470 nm light on a patch-clamped (whole cell configuration) myotube at −50 mV revealed a fast inward current that reached a maximum 3 ms after the end of the 4 ms light pulse (Fig. 2A). This fast transient inward current was followed by a deactivation slower than the activation with a duration between 30 and 70 ms depending on the light intensity. The relation between ChR2 amplitude and the light intensity is represented in Fig. 2B.

### Temporal light-excitation properties of ChR2-GFP expressing myotubes

**Figure 3:**
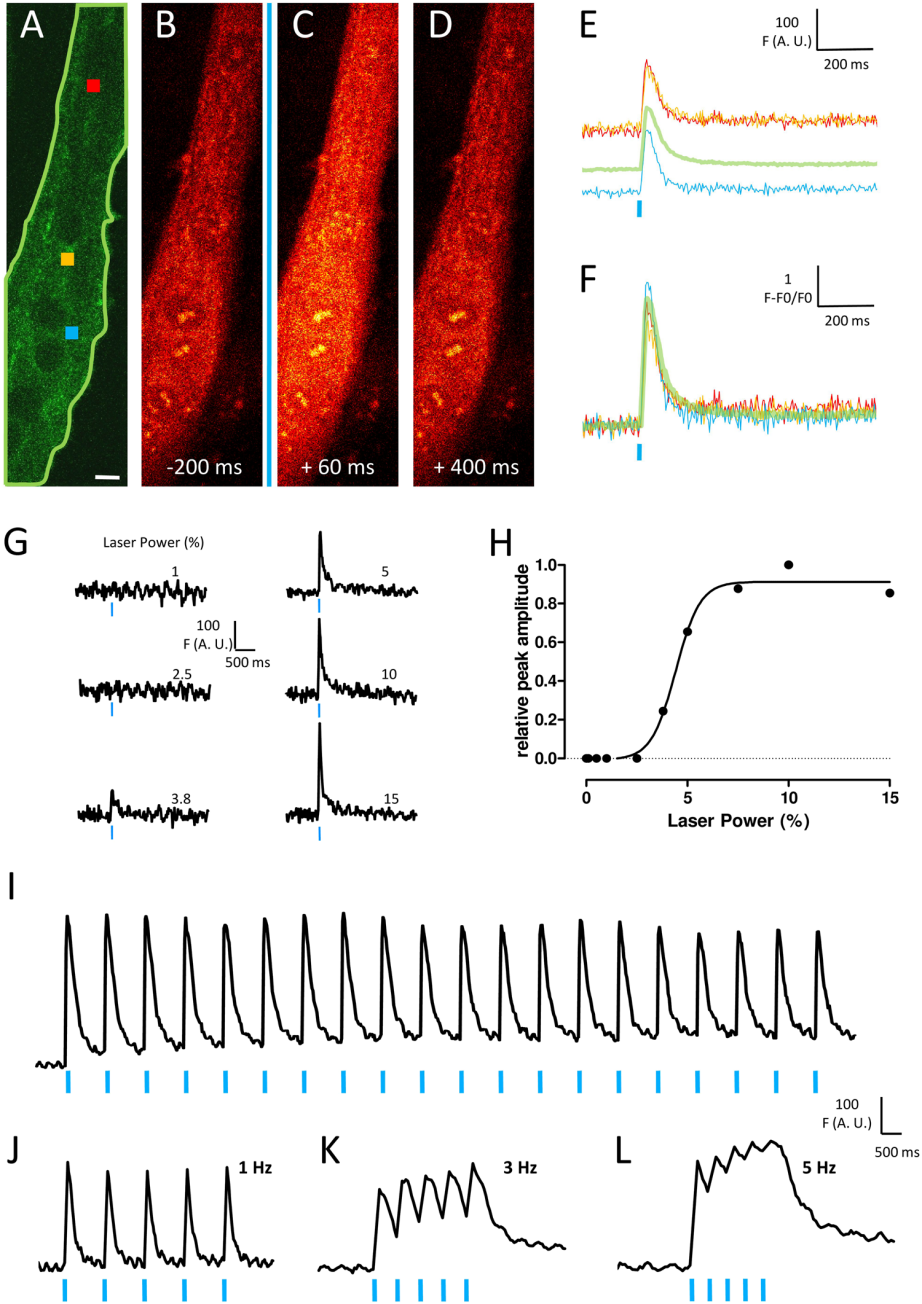
Temporal light-induced calcium response properties of ChR2-GFP expressing cells. (**A**) Several regions of interest were selected for measuring Rhod-2 fluorescence upon optical stimulation of ChR2-GFP expressing myotubes. The blue square represents the optical stimulation area and red and yellow squares display two regions of interest as examples. The light green line mark out the global region of interest where mean fluorescence was selected for measuring the global cell calcium transient. Bars: 10 µm. Series of fluorescence images were recorded and (**B**,**C** and **D**) are images selected 200 ms before, 60 ms and 400 ms after optical stimulation respectively. (**E**) row traces of fluorescence measured in region of interest described in (**A**). (**F**) Normalized same traces. (**G**) example of global fluorescence increases in a cell with increasing laser power of stimulation. (**H**) Relative peak amplitude of fluorescence as a function of laser power from the same experiment. (**I**) Rhod-2 fluorescence measurement during 20 repeated stimulations at 1 Hz. 5 repeated stimulations in a same cell at 1 Hz (**J**), 3 Hz (**K)** and 5 Hz (**L**). Vertical blue lines indicate the 4 ms light pulse.

In skeletal muscle, excitation-contraction coupling is a physiological mechanism that needs a strong and fast calcium transient to induce an appropriate Ca^2+^-dependent contraction. Light-evoked calcium transients in ChR2-GFP expressing myotubes were recorded to (i) know if the activation of a highly localized cluster of ChR2 channels was able to induce a large calcium increase in the entire cell, that is through the spreading of membrane depolarization and (ii) characterize spatial and temporal light-excitability properties of such a model. ChR2-GFP transfected C2C12 myotubes were loaded with high sensitive Rhod-2-AM calcium fluorescent probe used for confocal calcium imaging. Fast Rhod-2 calcium images (30 images/s) were recorded and ChR2-GFP cells were illuminated through the FRAP unit of the confocal microscope (Revolution, Andor, UK). Figure 3A–F shows the example of a ChR2-GFP myotube blue illuminated throughout a 5 × 5 µm area (blue square) and the resulting calcium increase in the whole investigated area (outlined by a green line in Fig. 3A). In this example, fluorescence signals recorded from different regions of interest (red and yellow squares, green outlined area in A and traces in E and F), showed similar kinetics. Various powers of illumination were also tested to estimate the excitability property of such a ChR2 cluster (Fig. 3G–H). From a same illumination area (5 × 5 µm), below a certain threshold power value (around 0.1 µW/µm^2^), no calcium increase was observed and above this value, the recorded calcium transients reached rapidly a maximum value corresponding to a maximal capacity of response to light-excitation. Furthermore, because native stimulation of muscle cells consists in nerve action potentials trains, calcium transients during repeated stimulations at various frequencies were measured. At 1 Hz, repeated light-stimulations induced repeated similar amplitude calcium transients (Fig. 3I), demonstrating that calcium increase and recovery mechanisms were not drastically altered during this protocol. By contrast, increasing the stimulation frequency, led to summation of calcium transients (Fig. 3K) which led eventually (Fig. 3L) to a “rough-tetanus”-like calcium signal, reaching a high intracellular calcium level. All these results are in agreement with the fact that ChR2 expression in differentiated muscle cells in culture allows their optical-stimulation without alterations of excitation-contraction coupling mechanism.

### Spatial light-excitation properties of ChR2-GFP expressing myotubes

**Figure 4:**
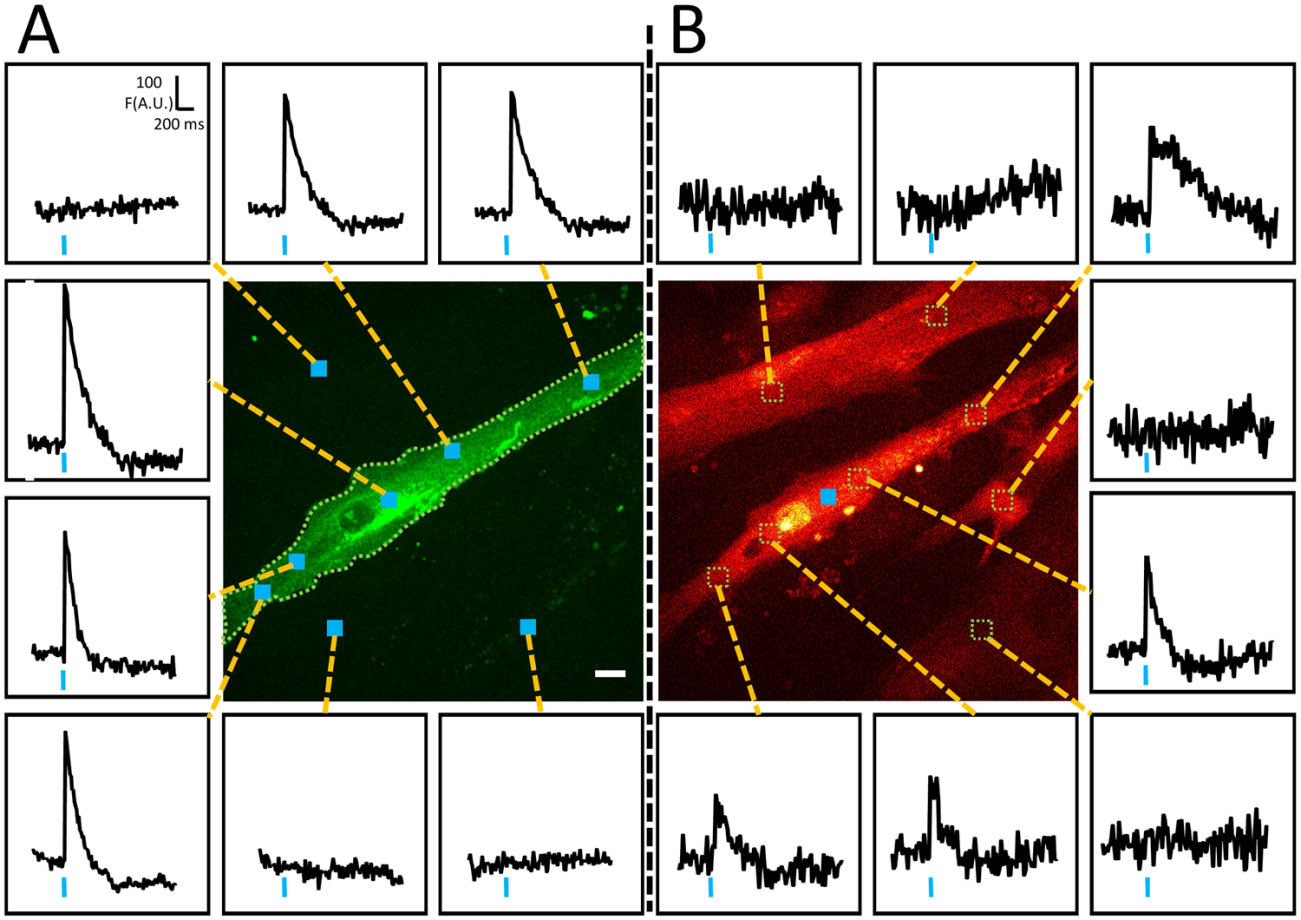
Spatial light-induced calcium response properties of ChR2-GFP expressing cells. (**A**) Global fluorescence records (insets) in the green dotted line outlined area of a ChR2-GFP expressing myotube depending on the position of the optical stimulation square (blue squares). (**B**) Local fluorescence records (insets) from regions of interest inside and outside the myotube (dotted lines squares) during an optical stimulation (blue square) inside the same myotube as in A. Bar: 10 µm.

At this development work step, three crucial points for our light-excitable muscle cells model had to be validated. First, it was important to know if the activation of different areas in the same interrogated cell led to a same global transient; second, check if stimulation of non ChR2 expressing neighboring cells did not induce response in tested cell, and third, check that stimulation of ChR2 expressing cells did not induce responses in neighboring cells. Figure 4 displays an example of such experiments in an optical field of myotubes culture. In this field, only one myotube was expressing ChR2-GFP (green). As expected, the optical activation of different areas (blue squares inside the myotube) of this myotube led to a similar calcium transient maximum amplitude, when recorded at the whole cell level (green dotted outlined area) (Fig. 4A). Conversely, illumination outside the ChR2-GFP expressing myotube did not induce any calcium transient (upper left panel traces and two lower right panel traces of Fig. 4A). Moreover (Fig. 4B), using the same area of illumination (blue square) in the myotube led to calcium increases in every regions of the myotube but had no effect on adjacent cells. Although it is beyond the scope of this paper, it is interesting to note that depending on the cell region, the shape of the calcium response was different. These observations show that our stimulation protocol is cell-selective and produces a light-evoked transient in the targeted cell.

### Activation of excitation-contraction coupling or of calcium-induced calcium release?

**Figure 5:**
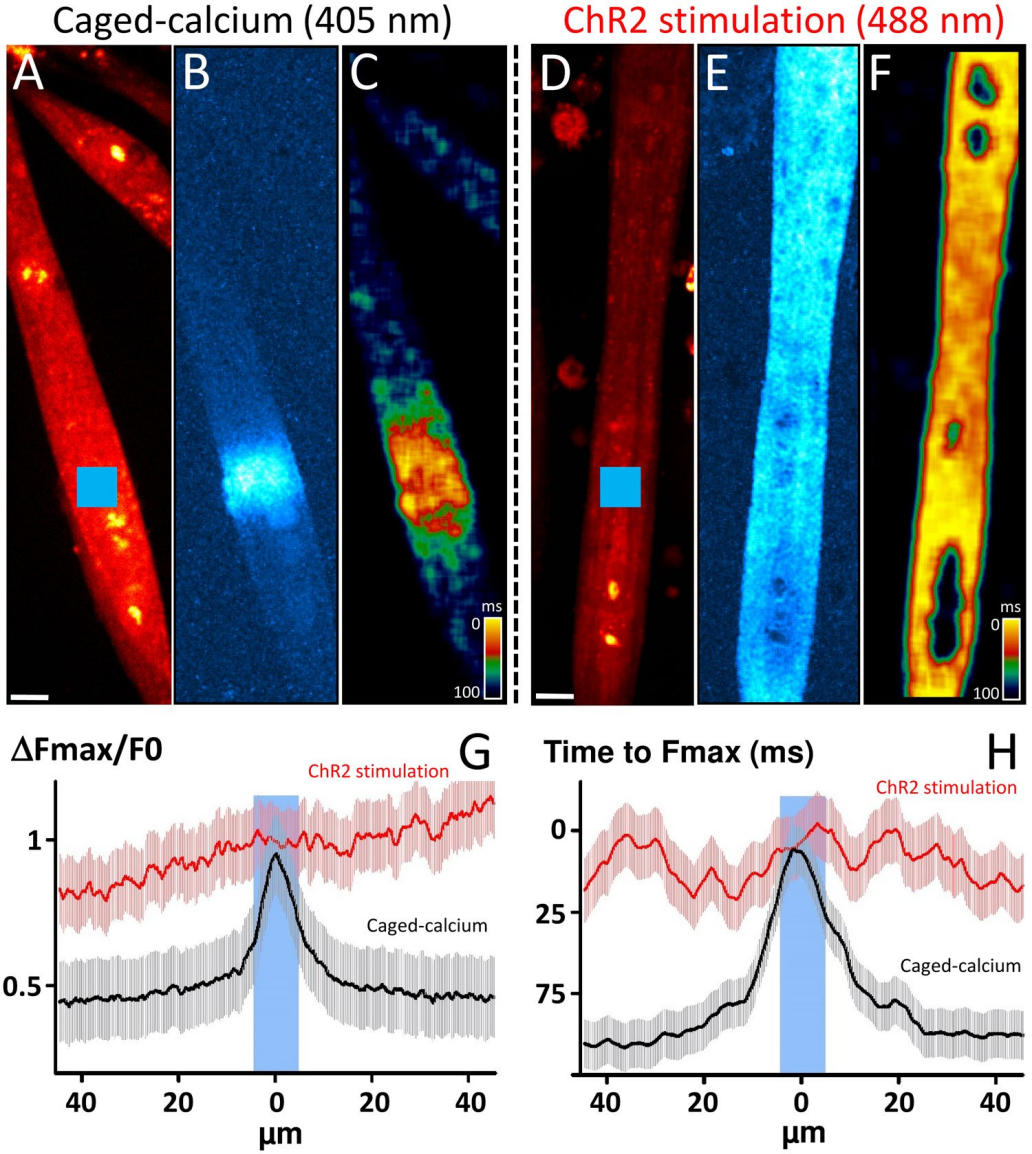
Comparison between Ca^2+^ increases induced by photorelease of caged-compound (uncaging) and by optogenetic stimulation. In each condition, time sequences of 350 fast confocal images (1 image every 24 ms) have been recorded in Rhod2–loaded myotubes. Bars: 10 µm. ***Uncaging***: Frames of fluorescence were obtained from C2C12 myotubes loaded with Rhod-2-AM and NP-EGTA-AM. (**A**) Image of mean fluorescence obtained in a C2C12 before flash. The blue square represents the area of photolysis (10 × 10 µm) with a 405 nm laser line. Bar, 10 µm. A single pulse was delivered during the trial. (**B**) Image computed from the image series and displaying the maximum of fluorescence, in the sequence, from each pixel normalized with the fluorescence before photolysis (ΔF/F0). (**D**) Image computed from the image series and displaying, in each pixel, the duration between the trial and the time needed to get the maximum of fluorescence obtained in (**B**). Inverted pseudo color scale displays therefore, in yellow, short durations, meaning a maximum obtained very early after photolysis and in blue longer durations, meaning a maximum obtained lately after photolysis. Hence, this image represents propagation properties of the calcium increase. ***Optogenetic stimulation***: Frames of fluorescence were obtained from ChR2-C2C12 myotubes loaded with Rhod-2-AM. (**D**) Image of mean fluorescence obtained in a C2C12 before light stimulation. The blue square represents the area of stimulation (10 × 10 µm) with a 488 nm laser line. Bar, 10 µm. A single pulse was delivered during the trial. (**E**) Image computed from the image series and displaying the maximum of fluorescence, in the sequence, from each pixel normalized with the fluorescence before photolysis (ΔF/F0). (**F**) Image computed from the image series and displaying, in each pixel, the duration between the trial and the time needed to get the maximum of fluorescence obtained in E. (**G**) Spatial representation of the relative maximum fluorescence measured from either side of the stimulation on several cells during the ChR2 stimulation (n = 12 cells) or Caged-calcium stimulation (n = 15 cells). (**H**) Spatial representation of the time to reach the maximum fluorescence around the stimulation on the same cells.

To better understand mechanisms that lead to calcium increase under optogenetic stimulation, we compared it to the calcium response obtained with flash photolysis of calcium caged in C2C12 myotubes at the same differentiation stage. Flash photolysis of caged compounds is a powerful technique for producing rapid changes in concentration of bioactive signaling molecules and uncaging can only occur in defined sub-cellular regions. Experiments were performed on C2C12 myotubes loaded with the Ca^2+^ indicator Rhod-2, and the Ca^2+^ cage NP-EGTA allowed to investigate kinetics and propagation of the calcium increase due to the release of calcium from its caged form in the cytoplasm (Fig. 5). Applying a brief and localized flash of 405 nm light (blue square in Fig. 5A) to a cell loaded with this “caged-Ca^2+^” produced a rapid jump in intracellular calcium concentration in the localized light area (Fig. 5B). Moreover, we calculated from the image series the time needed to get the maximum of fluorescence in each pixel and Fig. 5C displays these times values in each part of the myotube. This image indicates that, in this case, the propagation is restricted to a 20-µm area. The same protocol was performed in ChR2 expressing-C2C12 myotubes loaded with Rhod-2-AM, but with optical stimulation at 488 nm (blue square in Fig. 5D,E). The computed image representing propagation properties of the calcium increase shows clearly in this example that calcium increase affects quickly the entire cell in the same time (Fig. 5F). Spatial representation of the relative maximum fluorescence measured from either side of the stimulation was measured on several cells during the Caged-calcium stimulation (n = 15 cells) or ChR2 stimulation (n = 12 cells) and is represented in Fig. 5G. Figure 5C and F display spatial representation of the time to reach the maximum fluorescence around the stimulation. These results clearly show that optical ChR2 stimulation leads to a fast and strong calcium transient propagated in the entire stimulated cells in less than 25 ms (Fig. 5H). Therefore, this calcium increase is more the consequence of a fast membrane depolarization propagation rather than an intracellular calcium-induced calcium release process.

## Discussion

Excitation/calcium release coupling, which eventually leads to contraction, is a central mechanism of muscular function and is widely studied in the physiological and physiopathological context. C2C12 myotubes have previously been investigated as a model for muscle excitation-contraction coupling in several studies^24, 25^. These studies demonstrated that flux underlying voltage clamp-activated Ca^2+^ transients, first, consist mainly of voltage-controlled Ca^2+^ release from the sarcoplasmic reticulum and second, has similar characteristics as in primary cultured mouse myotubes and in mature fibers. Our work achieves a significant technical advance for muscular calcium study with the ability to induce and measure cell calcium responses following targeted optogenetic stimulation of restricted membrane areas.

Most studies employing ChR2 in cardiac and muscular optogenetic experiments have used a wide field of light stimulation to induce membrane depolarization^22, 23, 26^. However, by means of a 488 nm laser line, this fine and contactless control of membrane potential can be exploited to target stimulation in a restricted fraction of plasma membrane. In our experiments, shedding light for 4 ms on entire myotubes was sufficient to activate ChR2. However, we demonstrate that the stimulation of a small proportion of ChR2 through a targeted light stimulation to a small membrane area of 25 µm^2^ was sufficient to induce a global calcium increase response in the whole cell. Stimulation of area as small as 1 µm^2^ also allows the initiation of a calcium response depending on the laser intensity. By comparing this response to uncaging experiments, we observed that the calcium increase is more the consequence of a membrane depolarization propagation rather than a calcium increase propagation through a calcium-induced calcium release mechanism. Moreover, although the addition of TTX had no effect on the global light-induced calcium increase in myotubes (Fig. S2C), the addition of cadmium, which is known to block calcium influx but not calcium release from the sarcoplasmic reticulum^27, 28^ led to a local calcium increase, at the location of light-stimulation (Fig. S2F). This local increase leads us to think that activation of ChR2 in this area allowed a local membrane depolarization, which was strong enough to activate the ryanodine receptor opening through L-type calcium channels. Nevertheless, this depolarization did not allow sarcoplasmic reticulum release in the entire cell. Moreover, the addition of nifedipine completely abolished calcium increase (Fig. S2I). These results suggest that in our confocal imaging stimulation experiments, a local light-activation of ChR2 channels lead to a local depolarization that may be propagated in the entire cells through voltage gated Ca^2+^-channels.

Classical methods employed for investigating calcium responses associated to muscle membrane cell depolarization mainly used four types of technique: superfusion of a depolarizing medium, electrical field stimulation by means of large external wire electrodes, whole-cell depolarization through the patch pipette of the patch-clamp technique, and finally focal pressure application (“puff”) of acetylcholine. The first one involved superfusion of a medium containing high concentration of potassium (tens of mM K^+^) or a pharmacological compound like acetylcholine^29^. This method presents a low temporal resolution and a spatial non-homogeneity (time for K^+^ ions or compound to reach the whole membrane). In addition, by nature, this technique precludes short duration stimulations and fast recovery study. The second method^30^, using depolarization through voltage application by external wires, displays a good time resolution but a limited spatial homogeneity drastically depending on wires location and size relative to the cell shape and length, and to position of the particular fraction of membrane. The third method^24, 31^ relies on the whole-cell configuration of the patch-clamp technique and have an excellent temporal resolution. Nevertheless, this electrophysiological approach requires a complex implementation and precludes spatial studies as the whole cell membrane is depolarized. The fourth technique used for cell stimulation through the activation of excitation-contraction coupling is the focal pressure application (“puff”) of acetycholine through glass capillaries. Acetylcholine is loaded into a glass micropipette and the pipette tip (2–4 µm) is positioned at 40–50 µm from the cell^17^. Acetylcholine solutions are then pressure-ejected for a puff duration of commonly 5 to 50 s^18, 19^. This non-invasive approach is very interesting for stimulating cells with a native agonist and is close to the physiological excitation of muscle cells. Nevertheless, taking into account first, the distance between the tip and the cell and second, the time-scale of the puff duration, one can think that the entire cell (or a significant part of it) is superfused with acetylcholine for a non-physiological duration. One advantage of the optogenetic stimulation is that the stimulation area concerns the subcellular level with a stimulation duration of several milliseconds, which is close to the physiological excitation properties. Moreover, optogenetic stimulation allows also the application of non-invasive train of stimulations with various frequencies. This technical approach will allow to investigate the consequences of such stimulation on intracellular calcium dynamics.

In this context, our technique appears therefore to be the only one to present at the same time, the ability to perform fine localized electrical stimulations and calcium responses recordings, and a relatively easy implementation. This technique appears well adapted to study the calcium response induced by fast membrane depolarization. It allows repeated stimulation of a cell with the ability to induce a rough tetanus response. Moreover, this technique allows the safe, contactless, stimulation of a single cell within a culture dish and is therefore easily reproducible and not time-consuming in contrast to the stimulation by an electrical field or a superfusion system. Besides, the possibility to trigger a membrane depolarization in restricted membrane area is very interesting. Indeed, the stimulated area is similar or even narrower than the size of a neuromuscular junction^32^. Our experiments revealed that the stimulation of such a small restricted area enable to study the induced calcium response in another area of the cell. The first results obtained in our experiments revealed that the calcium response could change depending on the localization and, whereas this is beyond the scope of the present publication, we believe that this approach will allow exciting investigations in future.

The major limitation of our method relates with the temporal resolution which is limited to 40 fps on our setup. This value depends entirely on the performance of the imaging system. With the most sophisticated systems which recently appeared on the market or home-made, faster acquisition should be reached and faster calcium variation events addressed.

In conclusion, this study presents for the first time a method that allows optogenetic stimulations of muscle cells with fine temporal and spatial resolutions that mimic motor inputs. This powerful method is well adapted to the study of fast depolarization-induced intracellular calcium signaling and should bring new insight into the study of muscle calcium homeostasis.

## Methods

### Cell cultures and transfection

C2C12 cells, a mouse myogenic cell line, were maintained for no more than six passages at 37 °C with a 5% CO_2_ atmosphere in Dulbecco’s Modified Eagle’s Medium (DMEM), supplemented with 10% fetal bovine serum. C2C12 myoblasts were grown to 80–90% confluence on 35 mm-diameter tissue culture dishes with a bottom made from a glass coverslip, coated with matrigel (Corning, Bedford, USA) and subsequently transfected with pAAV-CAG-ChR2-GFP vector plasmids (Addgene #26929) using Lipofectamin 2000 transfection reagent (Invitrogen), according to the manufacturer’s instructions. Twenty-four hours post-transfection (Day 0), differentiation into multinuclear myotubes was promoted by switching to differentiation medium, i.e., DMEM containing 2% horse serum (Invitrogen).

### Electrophysiology

At day 6, ChR2 positive myotubes were identified using GFP fluorescence. The measurements were carried out at room temperature (≈22 °C). Patch electrodes (≈3 MΩ) were pulled from borosilicate glass capillaries using a vertical micropipette puller (Narishige, Tokyo, Japan). Experiments were performed using an Axopatch 200 A amplifier with a CV 202 AU headstage (Molecular Devices, CA, USA). Current variations were recorded using the whole-cell configuration of the patch-clamp technique. Resting membrane potential was estimated using I = 0 of the amplifier at the beginning of experiments. Voltage-clamp and light pulses were generated by a personal computer equipped with an analog-digital converter (Digidata 1322a, Molecular Devices) using pClamp software v10.2 (Molecular Devices). ChR2 currents were filtered and digitized at 5 KHz. For all the experiments a 5 s interval between each stimulations was applied. The digitized currents were stored on a computer for later off-line analysis. The patch pipettes were filled with (mM): 10 NaCl, 130 KCl, 0.5 MgCl_2_, 2 Mg-ATP, 1 EGTA and 10 HEPES. The pH was adjusted to 7.2 using KOH. The bath solution contained (mM): 140 NaCl, 5.4 KCl, 1.8 CaCl_2_, 1.8 MgCl_2_, 11 glucose, and 10 HEPES. The pH was adjusted to 7.4 using NaOH.

Cell illumination was performed through an optical fiber-coupled led of 470 nm (Thorlabs, Maisons-Lafitte, France) positioned close to the cell as would be a perfusion system. The led was connected to a DC2200 controller (Thorlabs, Maisons-Lafitte, France) driven through pClamp 10.2 to manage light pulses. Voltage-clamp experiments were performed at a holding potential of −50 mV with a 4 ms light pulse of various intensities (from 0.16 to 4.3 mW.mm^−2^). Analysis was performed using Clampfit 10.2. Light power was assessed by a power meter (Opton Laser Intern., Orsay, France) at the output of the optical fiber.

### Live confocal video microscopy

For ChR2 localization, 3D-fluorescence images were obtained in living cells stained with GFP with a laser scanning confocal microscope (Olympus FV1000, Japan). 488 nm line of argon laser was used for excitation of GFP and emission fluorescence was recorded through spectral detection channel between 490–590 nm (green fluorescence emission). Calcium uncaging experiments with 5 µM NP-EGTA-AM were performed using a 405 nm laser diode. 800 × 800 pixels images were acquired with UPLSAPO 60X W NA:1.20 objective lens. Numerical zooming to 0.13 µm pixel size was done in respect to oversampling Nyquist cryteria. 3D optical sectioning of 0.2 to 0.4 µm were driven with a step Z-axis motor. 3D images were analyzed with Imaris software (Bitplane, Switzerland).

Fast spinning disk confocal images were acquired using a Plan-APO 40X/1.25 NA objective on an Olympus IX81 microscope enclosed in a thermostatic chamber (Solent Scientist) coupled to an Andor Revolution Imaging System equipped with an EMCCD/iXon + -DU-897 camera coupled to a Sutter filter wheel and a Yokogawa CSU-X1. In these conditions, the optical section thickness was around 0.6 µm. IQ3 acquisition software (Andor technology) was used to acquire images of 512 × 512 pixels. For Ca2+ imaging, cells loaded with 5 µM Rhod-2-AM (Invitrogen) were illuminated at 561 nm. Emitted fluorescence was collected through a passband filter at 605 nm (30 nm width). Optical stimulation at 488 nm was performed with the use of the FRAP (Fluorescence after photobleaching) module of the Andor Revolution Imaging system (Andor, UK). These experiments were performed at 22 °C and in a standard bath solution as described for patch-clamp experiments. ImageJ (NIH) and IDL 6.3 were used to analyze and process data.

## Electronic supplementary material

Supplementary information
