# Supplementary material for: Optogenetic approach for targeted activation of global calcium transients in differentiated C2C12 myotubes
Source: Sci Rep. 2017 Sep 11;7:11108. doi: 10.1038/s41598-017-11551-z (PMC5593883; doi:10.1038/s41598-017-11551-z)
Supplement: Supplementary file 1 — Supplementary information [file 41598_2017_11551_MOESM1_ESM.pdf]

Supplementary information

Optogenetic approach for targeted activation of global calcium transients in differentiated C2C12 myotubes

Stéphane Sebille, Oualid Ayad, Charles-Albert Chapotte-Baldacci, Christian Cognard, Patrick Bois, Aurélien Chatelier*.

Laboratoire de Signalisation et Transports Ioniques Membranaires, Université de Poitiers, CNRS, 86073 POITIERS CEDEX 9, France.

*** Corresponding author :**
Aurélien Chatelier, Ph.D, Laboratoire de Signalisation et Transports Ioniques Membranaires, Université de Poitiers, CNRS, Pôle Biologie Santé,
1 rue Georges Bonnet, TSA 51106, 86073 POITIERS Cedex 9
Phone: +33 (0)5 49 45 37 47
Fax: +33 (0)5 49 45 40 14
E-mail: aurelien.chatelier@univ-poitiers.fr

Supplementary Figure S1 : Co-staining for ChR2 and mitochondria:

ChR2-C2C12 myotubes were stained for mitochondria using a specific incubation protocol of Rhod-2-AM (10 µM, 20', 30' wash, 37°c) that allows the dye accumulation in mitochondria[1]. **A**, **D**, **G**: green fluorescence of CHR2-GFP in ChR2-C2C12 myotubes. **B**, **E**, **H**: mitochondria staining. **C**,**F** and **I**: merge. **G**, **H** and **I** are magnified images from areas originating from **D**,**E** and **F,** and designed by the white rectangle in **F**. No apparent co-staining was observed. Similar staining patterns were observed with the mitochondrial JC-1 dye (data not shown).

Supplementary Figure S2 : Effect of channels blockers on light-evoked calcium transients :

Frames of fluorescence were obtained from ChR2-C2C12 myotubes loaded with Rhod-2-AM before and after the addition of TTX (10 µM), Cadmium (Cd^{2+}: 50 µM) and nifedipine (2 µM). **A**, **D**, **G**: images of mean fluorescence obtained in C2C12 myotubes before light stimulation. The blue square represents the area of stimulation with a 488 nm laser line. A single pulse was delivered during the trial. Bar, 10 µm. **B**-**I** : images computed from the image series and displaying the area under the curve value of fluorescence for 50 ms after stimulation, in the sequence, from each pixel, normalized with the fluorescence before stimulation (ΔF/F0). An instantaneous calcium increase was observed in the whole myotube before (**B**) and after the addition of TTX (**C**) (n=5). Only a local increase of calcium on the area of light stimulation was observed after the addition of Cd^{2+} (**F**) (n=2). No calcium increase was observed after the incubation of nifedipine (**I**) (n=3).

1. Molecular Probes, Product information : Rhod Calcium indicators. Mp012244.

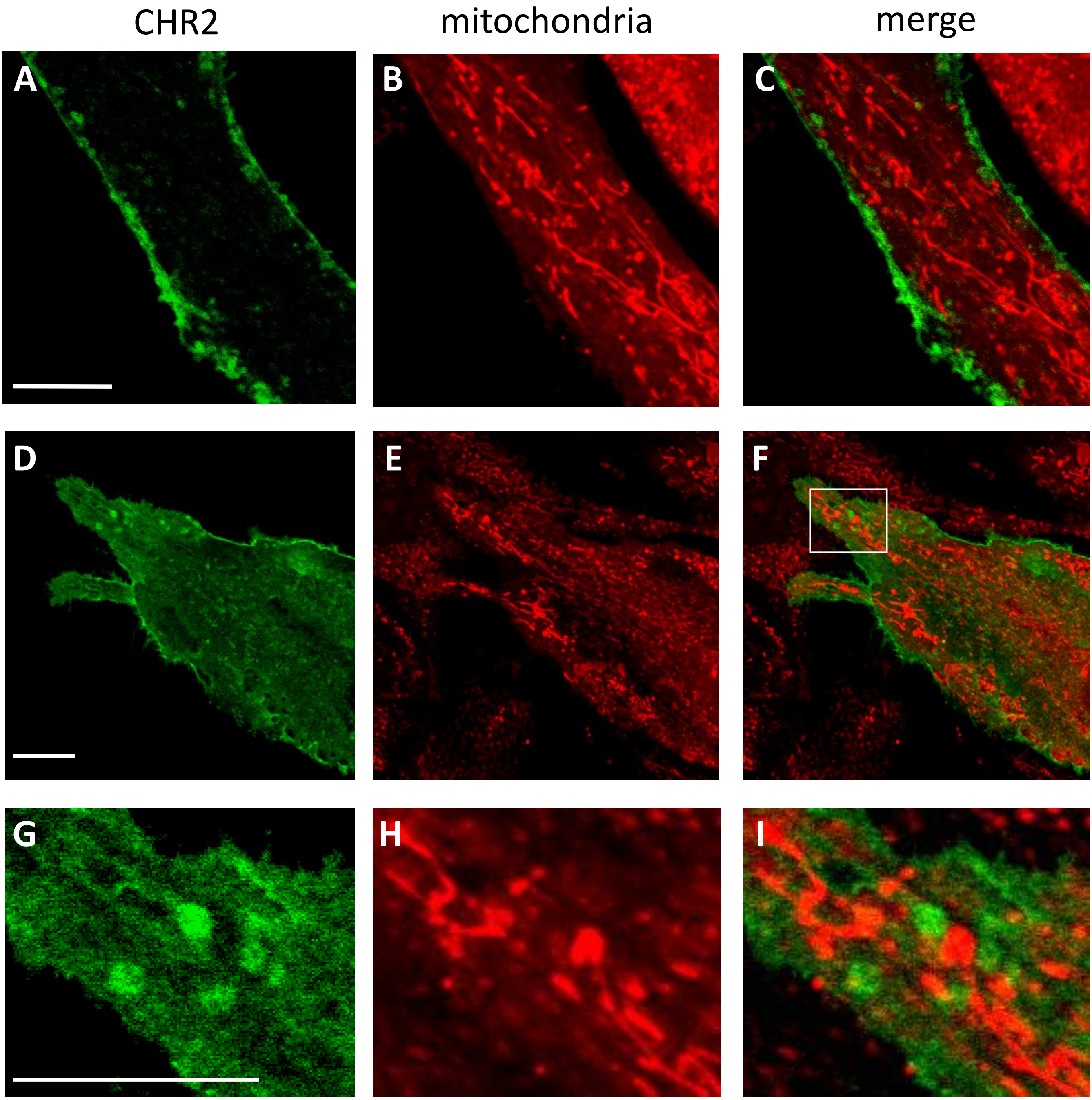

Supplementary Figure S1

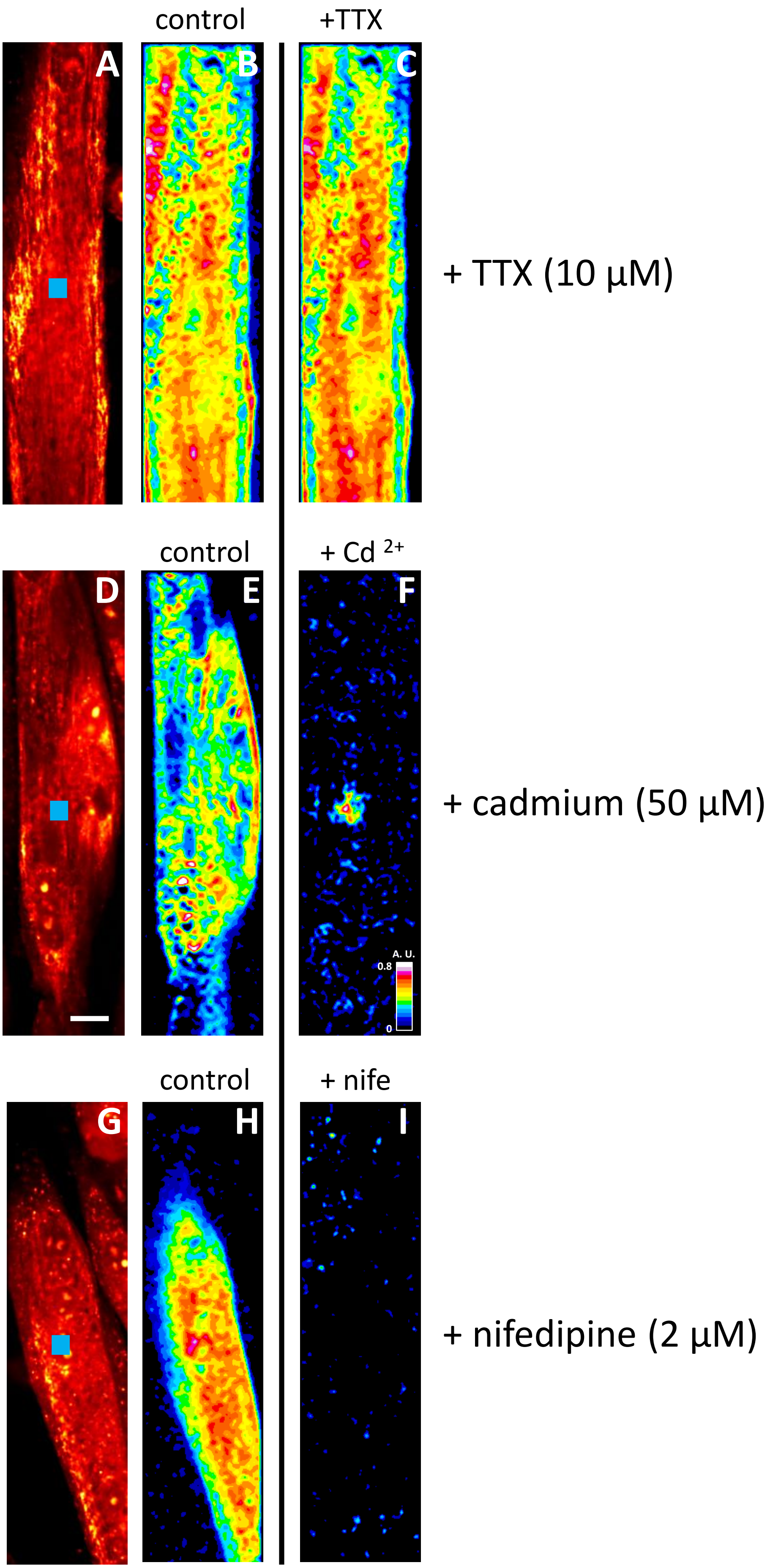

Supplementary Figure S2
